# Caring across Boundaries versus Keeping Boundaries Intact: Links between Moral Values and Interpersonal Orientations

**DOI:** 10.1371/journal.pone.0081605

**Published:** 2013-12-12

**Authors:** Laura Niemi, Liane Young

**Affiliations:** Department of Psychology, Boston College, Chestnut Hill, Massachusetts, United States of America; George Mason University/Krasnow Institute for Advanced Study, United States of America

## Abstract

Prior work has established robust diversity in the extent to which different moral values are endorsed. Some people focus on values related to caring and fairness, whereas others assign additional moral weight to ingroup loyalty, respect for authority and established hierarchies, and purity concerns. Five studies explore associations between endorsement of distinct moral values and a suite of interpersonal orientations: Machiavellianism, prosocial resource distribution, Social Dominance Orientation, and reported likelihood of helping and not helping kin and close friends versus acquaintances and neighbors. We found that Machiavellianism (Studies 1, 3, 4, 5) (e.g., amorality, controlling and status-seeking behaviors) and Social Dominance Orientation (Study 4) were negatively associated with caring values, and positively associated with valuation of authority. Those higher in caring values were more likely to choose prosocial resource distributions (Studies 2, 3, 4) and to report reduced likelihood of failing to help kin/close friends or acquaintances (Study 4). Finally, greater likelihood of helping acquaintances was positively associated with all moral values tested *except* authority values (Study 4). The current work offers a novel approach to characterizing moral values and reveals a striking divergence between two kinds of moral values in particular: caring values and authority values. Caring values were positively linked with prosociality and negatively associated with Machiavellianism, whereas authority values were positively associated with Machiavellianism and Social Dominance Orientation.

## Introduction

Across cultures and around the world people differ not only in what they take to be right or wrong but even in what they count as morally relevant at all [1]–[7]. Some people focus on the importance of individual rights, including the rights to be treated fairly and not harmed, whereas others focus additionally on moral norms that serve not lone individuals necessarily but entire communities. Specifically, concerns about caring for and not hurting or taking advantage of others are often designated as “individualizing” values [1], [2], . These norms are aimed at ensuring that each individual is protected. By contrast, concerns about being loyal to one's group, showing adequate respect for authority (and extant social structures, i.e. hierarchies), and maintaining bodily or spiritual purity often serve a different purpose – to maintain cohesive communities. Accordingly, “binding” values are thought to “bind and build” groups of people [1]–[3], [8].

Recently, however, researchers have suggested that “binding and dividing” [9] or “binding and blinding” [10] may reflect a better characterization of these values. On the one hand, moral communities guided primarily by binding values encourage their members to stay loyal to the group, to respect the relevant authorities, and to maintain community standards for spiritual and physical purity [11]. This represents the “bright” side of binding values. On the other hand, while group members may selflessly elevate the needs of their group above their own individual needs, they may also prioritize their own group over other groups (and other individuals in those different groups), leading to negative intergroup attitudes (e.g., prejudice, bias, condoning violence toward outgroups) [9], [12]–[15]. This represents the “dark” side of binding values and their tendency to blind and divide. Great strides have been made in psychological research to map moral values onto *political orientation* (e.g., links between binding values and conservative politics) [1], [2]; however, outstanding questions about the fundamental nature of various moral values highlight the need for further research that maps individuals' moral values onto *interpersonal orientations* (e.g., prosocial and antisocial tendencies). While moral values may be assumed to track with prosocial outcomes broadly (e.g., more moral values  =  moral advantage) [10], an outstanding empirical question is how people's “lofty” beliefs about right and wrong truly relate to more mundane, everyday interpersonal styles.

In fact, binding and individualizing values may be at odds with each other. Binding values concern the differences between groups (and individuals), whereas individualizing values can in principle motivate prosocial behavior across group boundaries. At the very least, nothing inherent to individualizing values dictates differential treatment across groups or individuals. Given the fundamental tension between binding and individualizing values and the presence of this tension in culture wars around the world [1], [4], [8], [16], [17], it is critical to examine empirically how these moral values relate to outcome variables that may matter for ordinary social relations. The approach we take here is to investigate whether individuals who endorse certain moral values also demonstrate other prosocial or antisocial tendencies, measured using independent and previously validated constructs [18], [19]. For example, are people who assign greater weight to binding values (e.g., valuation of authority) more Machiavellian and oriented toward social dominance? Do people who assign more weight to individualizing values (e.g., caring values) exhibit greater prosocial tendencies?

Most interpersonal behavior requires individuals to balance selfish motivation with prosocial motivation – to be a positive social partner who helps other people. These orientations are not mutually exclusive – care for the self is at times necessary to enable care for others. However, for some individuals, a motivation to dominate or exploit the group for selfish aims, measureable as Machiavellianism [20] or Social Dominance Orientation [21], may take precedence. Individuals high in Machiavellianism (“Machs”) admit to employing manipulation and deception to achieve power, status, control, and financial success [20]. These goals require successful management of group relations, which may in turn shed light on the paradoxical nature of Machiavellianism. Machs are often described as socially skilled, well-liked, popular, and excellent at building alliances [22], but they are also subclinically psychopathic [23] and exploitative of others' trust [24], [25]. Machiavellian negotiation of relationships and social structures for personal gain may benefit from a moral stance that elevates values like loyalty and deference to authority. More specifically, these values are critical for the preservation of existing social order but largely insensitive to concerns about caring and fairness. Moralization of these values – alongside relative indifference to caring and fairness values – could facilitate strategic hierarchy management while freeing the individual to feel morally justified in engaging in manipulative or exploitative behavior.

Relatedly, Social Dominance Orientation (SDO) is characterized by a desire for inequality and a tendency to categorize people along a hierarchical “superior-inferior dimension” [21]. SDO, like Machiavellianism, has been found to predict various antisocial outcomes, including explicit racism and sexism as well as reduced empathy and concern for others [21], [26], [27]. While SDO has previously been identified as negatively correlated with individualizing values and positively correlated with binding values [2], SDO has not yet received attention for its potential positive connection with binding values when political orientation is controlled. Since an orientation towards social dominance requires a strict hierarchical worldview, a positive correlation between SDO and authority values, regardless of political orientation, would be predicted [2].

In contrast to these antisocial interpersonal orientations, an individual may instead be motivated by a desire to be helpful or caring – a prosocial interpersonal orientation. This cooperative orientation involves the preference for equal distributions of resources between one's self and another (as measured, for instance, by the social values orientation task) [28]. In other words, prosocial individuals take a non-competitive stance that “levels the playing field.” Thus, values that warrant moral action only when certain conditions are present – a demand for loyalty, respect for authority, or adherence to purity norms, as in the case of the binding values – may be a poor fit with a more general prosocial interpersonal orientation across social contexts. Meanwhile, values related to unconditional caring and/or fairness may be better aligned with this orientation.

Previous research has demonstrated correlations between antisocial tendencies (namely, the “Dark Triad:” psychopathy, narcissism, and Machiavellianism) and typically conservative stances on a range of issues including capital punishment, the right to detain suspected terrorists indefinitely, and the right to wage war in defiance of UN resolutions [18]. While this research suggests that the typically conservative moral values (i.e., “binding values” – authority values in particular) that likely underlie such attitudes may likewise correlate with antisocial tendencies, this deeper connection has not yet been investigated. Moreover, the present research aims to discern the links between moral values and a more balanced set of interpersonal orientations, ranging from antisocial to prosocial. Furthermore, this research examines these connections both in the context of and independent of political orientation – a focal point of prior work.

In five studies, we characterized the relationships between moral values as measured by the Moral Foundations Questionnaire [2] (caring, fairness, ingroup loyalty, authority, purity) and interpersonal styles, in particular, Machiavellianism, Social Dominance Orientation, and prosocial resource distribution. Furthermore, to capture greater detail concerning the potential targets of individuals' prosocial behavior, we also assessed self-reported likelihood of helpful and unhelpful behaviors toward kin/close friends and acquaintances/neighbors. In Study 1, we examined associations between moral values and Machiavellianism. In Study 2, we investigated associations between moral values and prosocial resource distribution. In Study 3, we investigated the relationships observed in Studies 1 and 2 within a single paradigm. In Study 4, we again tested the relationships between moral values, Machiavellianism, and prosociality, in addition to Social Dominance Orientation and the reported likelihood of helpful and unhelpful behaviors toward different targets (e.g., kin/close friends versus neighbors/acquaintances). Study 5 used data from an unrelated study to again test the replicability of associations between Machiavellianism and moral values. Finally, although the correlational design of these studies precludes causal claims, meta-analyses were conducted to determine aggregated correlation coefficients, allowing for demonstration of the most robust relationships between moral values, Machiavellianism and prosociality observed across studies.

## Study 1: Machiavellianism and Morality

### Ethics Statement

The Boston College Institutional Review Board approved the ethics of all of the following studies. Informed consent was obtained from all participants using an online form.

### Study 1 Method

Study 1 tested the relationship between participants' endorsement of caring and fairness (i.e., individualizing values), and ingroup loyalty, authority, and purity values (i.e., binding values), and self-reported Machiavellian tendencies. Participants were 117 individuals (66 females, *M*_age_ = 34.71, *SD* = 11.23) who completed the study online via Amazon.com's Mechanical Turk for a small payment. An additional 15 participants were excluded for failing attention checks or for not completing the study. Criteria for attention-check exclusion for all studies was failure on the two catch questions provided in the MFQ (see Appendix S1 in File S1) or completion of a presented portion (8 items) of the MFQ in under 10 seconds, indicating inadequate time spent attending to, reading, and answering all questions. The main results of all studies were unchanged when analyses were conducted with no exclusions (see Appendix S6 in File S1).

Moral values were assessed using the 30-item Moral Foundations Questionnaire (MFQ; See Appendix S1 in File S1 for items) [2]. The five foundations (caring, e.g., “Compassion for those who are suffering is the most crucial virtue;” fairness, e.g., “Justice is the most important requirement for a society;” ingroup loyalty, e.g., “It is more important to be a team player than to express oneself;” authority, e.g., “If I were a soldier and disagreed with my commanding officer's orders, I would obey anyway because that is my duty;” and purity, e.g., “I would call some acts wrong on the grounds that they are unnatural.”) were examined separately. Machiavellianism was assessed using the Machiavellian Personality Scale (MPS: See Appendix S2 in File S1 for items) [20]. The MPS contains four subscales: (1) amorality (endorsement of lying, cheating, e.g., “I believe that lying is necessary to maintain a competitive advantage over others”), (2) control (e.g., “I enjoy having control over other people”), (3) status (e.g., “I want to be rich and powerful someday”), and (4) distrust (e.g., “Other people are always planning ways to take advantage of the situation at my expense”). Participants completed additional survey questions unrelated to the main hypotheses, which followed all dependent measures reported here (see File S1). Finally, participants completed questions about their age, sex, political orientation, and religiosity. We note that Studies 1–3 presented scales in a fixed order (Study 1: MFQ, MPS; Study 2: MFQ, SVO; Study 3: MFQ, SVO, MPS; the order of items within scales was randomized). The testing of the various measures within the same session may have introduced pressure for participants to be consistent with their responses. This, however, appears to be less of a concern for links between moral values and Machiavellianism compared to caring values and prosocial resource distributions – values centered on universal caring share an intuitive connection with prosociality, whereas connections between moral values and Machiavellianism may be counterintuitive.

Our primary analyses involved first computing zero-order correlations to determine the direct relationships between the moral values tested and Machiavellianism. Next, partial correlations were computed, controlling for gender, political orientation (using a 7-point scale from “Very conservative” to “Very liberal”), and religiosity (using a 7-point scale from “Not at all religious” to “Very religious”). All correlations are reported in Table 1, 1a.

**Table 1:** Moral values and Machiavellianism: Correlations across Studies 1, 3, 4, 5.

	Mach Amorality (Partial)	Mach Control (Partial)	Mach Status (Partial)	Mach Distrust (Partial)	Mach Total (Partial)
1a) Study 1: n = 117					
**Caring**	−.235* (−.169)	−.231* (−.158)	−.043 (.051)	−.019 (.041)	−.165 (−.070)
**Fairness**	−.164 (−.121)	−.112 (−.052)	.024 (.100)	.042 (.090)	−.065 (.010)
**Ingroup**	.218* (.294**)	.072 (.102)	.417*** (.458***)	.256** (.297***)	.323** (.394***)
**Authority**	.113 (.235*)	−.018 (.030)	.293** (.362***)	.199* (.256**)	.203* (.308***)
**Purity**	−.063 (.065)	−.049 (.033)	.075 (.144)	.082 (.158)	.019 (.140)
1b) Study 3: n = 115					
**Caring**	−.351*** (−.234*)	−.255** (−.188*)	−.235* (−.170)	−.190* (−.122)	−.324*** (−.223*)
**Fairness**	−.189* (−.105)	−.306*** (−.279**)	−.098 (−.051)	−.114 (−.058)	−.210* (−.141)
**Ingroup**	−.013 (.028)	.160 (.136)	.137 (.111)	.013 (.021)	.079 (.084)
**Authority**	−.039 (.029)	.108 (.106)	.164 (.153)	.036 (.043)	.074 (.098)
**Purity**	−.022 (.048)	−.043 (.019)	.142 (.124)	.069 (.073)	.071 (.087)
1c) Study 4: n = 117					
**Caring**	−.216* (−.215*)	−.263** (−.238*)	−.390*** (−.381***)	−.011 (−.035)	−.279** (−.277**)
**Fairness**	−.121 (−.167)	−.137 (−.120)	−.193* (−.188*)	.012 (.011)	−.138 (−.148)
**Ingroup**	−.117 (−.037)	−.040 (−.044)	.043 (.058)	.029 (.007)	−.029 (−.003)
**Authority**	.071 (.207*)	.058 (.060)	.202* (.241**)	.120 (.102)	.156 (.213*)
**Purity**	−.149 (.004)	−.033 (−.066)	.051 (.064)	.212* (.169)	.038 (.076)
1d) Study 5: n = 187					
**Caring**	−.287*** (−.219***)	−.007 (−.041)	−.103 (−.017)	−.129 (−.054)	−.194** (−.097)
**Fairness**	−.022 (.011)	.031 (.052)	−.060 (−.014)	.020 (.032)	−.011 (.059)
**Ingroup**	−.115 (−.031)	.185* (.197**)	.328*** (.347***)	.090 (.115)	.146* (.199**)
**Authority**	−.076 (.044)	.093 (.106)	.313*** (.352***)	.187* (.228**)	.174* (.253***)
**Purity**	−.196** (−.027)	.053 (.108)	.155* (.244***)	.178 (.172*)	.023 (.169*)

**Notes.** “Partial” refers to partial correlations with political orientation, religiosity, and gender controlled. Zero-order correlation coefficient is presented first, partial correlation coefficient is in parentheses. Boldface indicates significant correlations. * *p*<.05, **p<.01, ***p<.001.

### Study 1 Results and Discussion

Correlations with demographic variables are reported first. Replicating prior work, ingroup loyalty, authority, and purity values (i.e., binding values) were associated with religiosity (*r* = .353, *r* = .404, *r* = .584, *p*'s<.001, respectively) and conservative political orientation (*r* = −.315, *r* = −.363, *r* = −.358, *p*'s<.001) [2]. By contrast, caring and fairness values (i.e., individualizing values) were associated with liberal political orientation (*r* = .325, *r* = .319, *p*'s<.001) and not with religiosity (*p*'s>.39). Female gender was associated with caring, authority, and purity values (*r* = .277, *p* = .002; *r* = .195, *p* = .035; *r* = .216, *p* = .019). We had no prior hypotheses about the association between female gender and moral values, and this association emerged as inconsistent across studies (see results of Studies 2, 3, and 4). We do not discuss gender differences further.

Table 1 (1a) displays the zero-order correlations between the Machiavellianism (Mach) Total score and Mach subscale scores and moral values (i.e., caring, fairness, ingroup loyalty, authority, and purity values). We found that the Mach Total score correlated positively with ingroup loyalty (*r* = .323, *p*<.001) and authority (*r* = .203, *p* = .029) values. We then examined each of the Mach subscales (i.e., amorality, control, status-seeking, distrust) separately. Mach Amorality was negatively associated with caring values (*r* = −.235, *p* = .011) and positively associated with ingroup loyalty values (*r* = .218, *p* = .018). Similarly, Mach Control was negatively associated with caring values (*r* = −.231, *p* = .012). Mach Status-Seeking and Mach Distrust were both also positively associated with ingroup loyalty (*r* = .417, *p*<.001; *r* = .256, *p* = .005, respectively) and authority (*r* = .293, *p*<.001; *r* = .199, *p* = .031, respectively) values.

We report partial correlations, controlling for any effects of gender, politics, and religion, in Table 1 (1a) as well. Links between caring values and Mach scale scores dropped below significance. The associations between Mach Total score and ingroup loyalty (*r* = .394, *p*<.001) and authority (*r* = .308, *p*<.001) values remained significant. Similarly, Mach Amorality, Status-Seeking, and Distrust remained significantly correlated with ingroup loyalty (Amorality: *r* = .294, *p* = .002; Status-Seeking: *r* = .458, *p*<.001; Distrust: *r* = .297, *p* = .001) and authority (Amorality: *r* = .235, *p* = .012; Status-Seeking: *r* = .362, *p*<.001; Distrust: *r* = .256, *p* = .006) values.

In sum, Study 1, in addition to replicating prior associations among moral values, religiosity, and political orientation [2], reveals negative zero-order correlations between caring values and Mach Amorality and Mach Control, and positive zero-order and partial correlations (controlling for religiosity, gender, and politics) between ingroup loyalty and authority values and Machiavellianism – particularly the Status-Seeking, Distrust, and Amorality subscales. We explore these associations in subsequent studies.

Due to the emergence of positive correlations between some moral values and Machiavellianism – an interpersonal orientation with *antisocial* characteristics – we next examined whether moral values would differentially track with a *prosocial* interpersonal orientation in Study 2.

## Study 2: Prosociality and Morality

### Study 2 Method

Study 2 provided an initial investigation of the relationship between different moral values and participants' preferences for prosocial resource distributions using the social values orientation task (SVO) [28]. Participants were 112 individuals (69 females, *M*_age_ = 34.40, *SD* = 12.61) who completed the study online as in Study 1; an additional 12 participants were excluded. Moral values were assessed as in Study 1.

Resource distribution preferences were established using a previously validated social values orientation task [28]. This task asked participants to select one of three different ways of distributing points to the self versus an unknown “other”: (1) *prosocial* choices delivered equal payouts to self and other (e.g., Self: 500/Other: 500), (2) *individualistic* choices maximized one's own benefit without concern that the other would receive less (e.g., Self 560/Other: 300), and (3) *competitive* choices minimized payout to the other even though the choice was also costly to the self (e.g., Self: 480/Other: 80). As in prior work [28]–[31], participants were advised that they were to imagine the “other” as a random person they would not meet in the future. Instructions noted that there were no right or wrong answers, and that the points had value – “The more of them you accumulate the better for you. Likewise, from the other's point of view, the more points s/he accumulates, the better for him/her.” Following the procedures of prior research [29]–[31], we took the number of prosocial choices as our key measure of prosociality. As in Study 1, participants completed questions about their age, sex, political orientation, and religiosity. Zero-order and partial correlational analyses were conducted (reported in Table 2, 2a).

**Table 2:** Moral values, prosociality, and Social Dominance Orientation: Correlations across Studies 2, 3, and 4.

	Prosociality (Partial)	SDO (Partial)
2a) Study 2: n = 112		
**Caring**	.202* (.121)	
**Fairness**	.137 (.109)	
**Ingroup**	.008 (−.021)	
**Authority**	−.067 (−.093)	
**Purity**	.013 (−.038)	
2b) Study 3: n = 115		
**Caring**	.227* (.164)	
**Fairness**	.241** (.210*)	
**Ingroup**	.035 (.056)	
**Authority**	−.040 (−.028)	
**Purity**	−.043 (−.023)	
2c) Study 4: n = 117		
**Caring**	.188* (.214*)	−.415*** (−.346***)
**Fairness**	.095 (.136)	-.495*** (−.414***)
**Ingroup**	.100 (.053)	.275** (.154)
**Authority**	−.006 (−.081)	.416*** (.279**)
**Purity**	.122 (.050)	.204* (−.009)

**Notes.** “Partial” refers to partial correlations with political orientation, religiosity, and gender controlled. Zero-order correlation coefficient is presented first, partial correlation coefficient is in parentheses. SDO =  Social Dominance Orientation. Boldface indicates significant correlations. * *p*<.05, ** *p*<.01, *** *p*<.001.

### Study 2 Results and Discussion

As in Study 1 and prior work, ingroup loyalty, authority and purity values (i.e., binding values) were associated with conservative political orientation (*r* = −.228, *p*<.016, *r* = −.346, *p*<.001, *r* = −.385, *p*<.001, respectively) and religiosity (*r* = .304, *r* = .378, *r* = .571, *p*'s<.001). Fairness values were associated with liberal politics (*r* = .189, *p* = .046). Gender (female) was also associated with caring values (*r* = .337, *p*<.001).

As shown in Table 2 (2a), we observed a zero-order positive correlation between prosociality and caring values (*r* = .202, *p* = .033), though this association emerged as a non-significant trend when controlling for gender, politics and religiosity. No associations were observed between prosociality and the other moral values we examined. Study 3 below provides a further investigation of the observed trend between caring values and prosociality while again investigating the links between moral values and Machiavellianism found in Study 1.

## Study 3: Machiavellianism, Prosociality, and Morality

### Study 3 Method

Study 3 aimed to replicate the key result of Study 1 (i.e., the relationship between Machiavellianism and ingroup loyalty and authority values) and also to follow up on the trend observed in Study 2 (i.e., the relationship between prosociality and caring values), within a single paradigm. Participants were 115 individuals (63 females, *M*_age_ = 38.33, *SD* = 11.82) who completed the study online as in the prior studies; an additional 7 participants were excluded. Study 3 used the Moral Foundations Questionnaire (MFQ) [2] as in Studies 1–2, the Machiavellian Personality Scale (MPS) [20] as in Study 1, and the social values orientation task [28] as in Study 2. Participants completed additional surveys not reported here related to measures of guilt and shame (see File S1 for Method and Results; Table S1 in File S1; and Appendix S5 in File S1). Finally, as in Studies 1–2, participants completed questions about their age, sex, political orientation, and religiosity. The same correlational analyses used in Studies 1–2 were conducted on the data collected in Study 3 (reported in Table 1 (1b) and Table 2 (2b).

### Study 3 Results and Discussion

As in Studies 1–2 and prior work, ingroup loyalty, authority, and purity values (i.e., binding values) were associated with conservative political orientation (*r* = −.199, *p* = .033; *r* = −.324, *p*<.001; *r* = −.453, *p*<.001) and religiosity (*r* = .358, *r* = .511, *r* = .580, *p*'s<.001). Caring and fairness values (i.e., individualizing values) were associated with liberal political orientation (*r* = .278, *r* = .280, *p*'s<.01) and female gender (*r* = .374, *p*<.001; *r* = .226, *p* = .015).

#### Moral Values and Machiavellianism

As shown in Table 1 (1b), zero-order correlational analysis showed that Mach Total score was negatively associated with both caring values (*r* = −.324, *p*<.001) and fairness values (*r* = −.210, *p* = .024). These links with Machiavellianism were driven primarily by negative associations with Mach Amorality and Mach Control (*p*'s*<*.05), as in Study 1, but the same effects held for Mach Status-Seeking and Mach Distrust (*p*'s<.05) for caring values. Unlike Study 1, significant positive zero-order or partial correlations were not observed between Machiavellianism and ingroup loyalty or authority values. However, positive trends were observed between Mach Status-Seeking and authority values (*p* = .08), and also between Mach Control and ingroup loyalty values (*p* = .08).

To review the findings related to Machiavellianism thus far, we found in Study 1 that (1) Machiavellianism was negatively associated with caring values and positively associated with ingroup loyalty and authority values, and (2) associations between Machiavellianism and ingroup loyalty and authority values remained significant when controlling for gender, religiosity, and politics. Here, in Study 3, we found similar but non-significant associations between ingroup loyalty and authority values and Machiavellian tendencies (Control, Status-Seeking), whereas caring values emerged as negatively associated with Machiavellianism broadly (Mach Total, Amorality, Control, Status-Seeking, Distrust), in both zero-order and partial correlations.

#### Moral Values and Prosociality

As in Study 2, a zero-order correlation (see Table 2, 2b) was observed between caring values and prosociality (*r* = .227, *p* = .015). Additionally, fairness values were also positively correlated with prosociality (*r* = .241, *p* = .010). Partial correlations controlling for gender, politics, and religiosity revealed that the relationship between fairness values and prosociality remained significant (*r* = .210, *p* = .027). Again, as in Study 2, no correlations were observed between the other tested moral values and prosociality.

#### Study 3 Conclusions

To summarize, we found that the significant zero-order and partial correlations observed in Study 1 between Machiavellianism and ingroup loyalty and authority values emerged as non-significant trends in Study 3. In contrast, the negative zero-order correlations observed between caring values and aspects of Machiavellianism in Study 1 were found broadly across both zero-order and partial correlations here in Study 3. We aimed to resolve these discrepancies by conducting a new study, Study 4. In addition, we added a measure of Social Dominance Orientation (SDO) [21], an interpersonal orientation that, like Machiavellianism, involves pursuit of dominance over others and rejection of equality. As SDO has been linked with antisocial tendencies (e.g., racism, sexism, low empathy) [21], [26], [27], we expected the inclusions of SDO would help to clarify our characterization of the tested moral values. Previously, SDO has been found to be negatively correlated with individualizing values and positively correlated with binding values [2]. Replication of these links in a new dataset alongside tests of Machiavellianism and prosociality would provide additional validation for our methods. Moreover, links between SDO and moral values have been unexplored when controlling for key variables of political orientation, religion and gender.

## Study 4: Machiavellianism, Social Dominance Orientation, Prosociality, and Morality

### Study 4 Method

Participants were 117 individuals (59 females, *M*_age_ = 36.37, *SD* = 12.99) who completed the study online as in the prior studies; an additional 12 participants were excluded. Study 4 again tested the relationship between participants' endorsement of moral values (MFQ) [2] and reported Machiavellian tendencies (MPS as in Studies 1 and 3) [20], given discrepancies between Studies 1 and 3. Study 4 also assessed Social Dominance Orientation using the 16-item Social Dominance Orientation Scale (SDO: See Appendix S4 in File S1 for items) [21]: participants rated the extent of their agreement with statements about equality and social dominance (e.g., “If certain groups stayed in their place, we would have fewer problems”; “We should do what we can to equalize conditions for different groups”).

In addition to administering the social values orientation task (i.e., the prosociality measure used in Studies 2 and 3) [28], we also constructed a novel task to measure participants' attitudes toward “helping” behaviors (See Appendix S3 in Supporting Information File S1 for all items; see also Table 3 for examples) to obtain a more detailed understanding of the relationship between prosocial or helping behaviors and moral values. Specifically, whereas the social values orientation task [28] involves distributing points between the self and an anonymous “other,” our novel helping task was designed to investigate whether links between moral values and prosociality would vary if a prosocial act was targeted at a person who was in a close versus a distant relationship with the giver. In this new task, participants rated the likelihood of their own helping behaviors directed at kin/close friends versus acquaintances, across four short scenarios (the order of which was randomized across participants). Participants read four scenarios describing hypothetical helping behaviors (one of each: picking up paperwork, giving ride to airport, moving branches, storing a bureau) in a 2×2 design such that the favor could be for (a) kin/best friend, or (b) for an acquaintance. Each participant received four scenarios in which (1) the protagonist *helped* kin/best friend, (2) the protagonist *did not help* kin/best friend, (3) the protagonist *helped* an acquaintance, and (4) the protagonist *did not help* an acquaintance. The scenarios presented were randomly selected from four possible kin/best friend scenarios and four possible acquaintance scenarios that did not repeat in content. Acquaintances included a man who worked next door, a woman who lived nearby, a neighbor down the street, a neighbor who just moved in nearby; kin were represented by either a brother or a mother. After each scenario, participants rated the likelihood that they would have acted as the protagonist did, using a 7-point Likert scale from “Not at all likely” to “Very likely”.

**Table 3:** Examples of helping task items in Study 4.

	Helping	Not Helping
**Kin/Close Friend**	Lisa's best friend asks Lisa if she will let her store a bureau in her basement for a couple months. Lisa decides to let her store the bureau in her basement.	Lisa's best friend asks Lisa if she will let her store a bureau in her basement for a couple months. Lisa decides to not let her store the bureau in her basement.
**Acquaintance**	A woman who lives nearby asks Lisa if she will let her store a bureau in her basement for a couple months. Lisa decides to let her store the bureau in her basement.	A woman who lives nearby asks Lisa if she will let her store a bureau in her basement for a couple months. Lisa decides to not let her store the bureau in her basement.

We hypothesized that self-reported likelihood of helping distant others would be associated with caring values, as these values are not theoretically constrained by the identity of the target. By contrast, the possibility that helping close others would be linked with binding values is supported by the characterization of binding values as moral values aimed at binding individuals together in tight-knit communities [1], [2], [8], [32].

Participants completed the helping task first, followed in random order by the SDO, SVO, MPS and MFQ. Finally, as in Studies 1–3, participants completed questions about their age, sex, political orientation, and religiosity. As in Studies 1–3, zero-order and partial correlational analyses were used (reported in Tables 1c, 2c, and 4). We also conducted a 2 (kin/close friends, acquaintances) ×2 (help, not help) ANOVA to examine the reported likelihood of helping versus not helping across different targets.

**Table 4:** Correlations between helping task items and moral values in Study 4.

	Help Kin (Partial)	Not Help Kin (Partial)	Help Acquaint. (Partial)	Not Help Acquaint. (Partial)
**Caring**	.079 (.066)	−.207* (−.210*)	.256** (.318***)	−.195* (−.230*)
**Fairness**	.135 (.133)	−.210* (−.199)	.184* (.254**)	−.100 (−.139)
**Ingroup**	−.011 (−.012)	−.030 (−.052)	.232* (.191*)	−.162 (−.131)
**Authority**	.030 (.034)	−.120 (−.162)	.096 (.026)	−.078 (−.031)
**Purity**	.101 (.131)	−.106 (−.167)	.270** (.268**)	−.144 (−.112)

**Notes.** “Partial” refers to partial correlations with political orientation, religiosity, and gender controlled. Zero-order correlation coefficient is presented first, partial correlation coefficient is in parentheses. Boldface indicates significant correlations. * *p*<.05, ** *p*<.01, *** *p*<.001.

### Study 4 Results and Discussion

As in Studies 1–3 and prior work, ingroup loyalty, authority, and purity values (i.e., binding values) were associated with conservative political orientation (*r* = −.325, *r* = −.428, *r* = −.505, *p*'s<.001) and religiosity (*r* = .188, *p = *.043, *r* = .245, *p = .*008, r = .494, *p*<.001). Caring and fairness values (i.e., individualizing values) were associated with liberal political orientation (*r* = .196, *p* = .035; *r* = .281, *p* = .002). Female gender was associated with caring values (*r* = .246, *p* = .008).

#### Moral Values and Machiavellianism

As shown in Table 1 (1c) and aligning with Study 3, zero-order correlational analysis showed that caring values were negatively associated with Mach Total score (*r* = −.279, *p* = .002) and several subscales: Mach Amorality, Mach Control, and Mach Status-Seeking (*p*'s<.05). Mach Status-Seeking was also negatively associated with fairness values (*p*<.05). Like Study 1, a positive zero-order correlation was observed between Mach Status-Seeking and authority values (*r* = .202, *p* = .029). Partial correlations controlling for religiosity, politics and gender showed that caring values remained negatively associated with Machiavellianism (Mach Total, Amorality, Control, Status-Seeking: *p*'s<.05); and significant positive associations emerged between authority values and Mach Total, Amorality, and Status-Seeking (*p*'s<.05).

#### Summary: Machiavellianism and Morality

To summarize the results of Studies 1, 3, and 4 (Table 1: 1a, 1b, and 1c) related to Machiavellianism and moral values, we saw that in zero-order correlations across all three studies, *caring values* were negatively associated with Machiavellianism. When the effects of religion, politics and gender were partialled out, these links remained significant in both Studies 3 and 4 (Table 1: 1b and 1c). Thus, there appear to be reliable negative associations between caring values and Machiavellianism – primarily Mach Amorality, Control and Status-Seeking. By contrast, we saw in both Studies 1 and 4 (Table 1: 1a and 1c) (and as a trend in Study 3: Table 1: 1b), a reliable *positive* association between Mach Amorality and Status-Seeking and *authority values*. The *positive* links between authority values and Machiavellianism on the one hand and the *negative* links between caring values and Machiavellianism on the other hand are echoed by the associations among these moral values and Social Dominance Orientation, reported in the next section.

#### Moral Values and Social Dominance Orientation

As shown in Table 2 (2c), zero-order correlational analysis revealed that caring and fairness values (i.e., individualizing values) were negatively correlated with Social Dominance Orientation (*p*'s<.001) [2]. By contrast, ingroup loyalty, authority, and purity values (i.e., binding values) were *positively* associated with Social Dominance Orientation (*p*'s<.05). Interestingly, partial correlations controlling for the effects of religiosity, politics and gender showed that negative links between SDO and both individualizing values held, while, among the binding values, only authority values remained significantly positively associated with SDO. This represents a novel demonstration of links between SDO and moral values, regardless of political orientation, as well as an instance when the binding values do not track together; we return to this point in the General Discussion. In Studies 1 and 4, authority values were most reliably positively linked with Machivellianism and, in particular, with Mach Status-Seeking. Together, these results suggest a link between moral values related to authority and interpersonal orientations that involve support of hierarchical social structures, which may maintain inequality and division between groups.

#### Moral Values and Prosociality

As in Studies 2 and 3, a zero-order correlation (Table 2: 2c) was observed between caring values and prosociality as measured by the social values orientation task (*r* = .188, *p = *.042). Partial correlations controlling for gender, politics, and religiosity showed that the relationship between caring values and prosociality remained significant (*r* = .214, *p* = .022). Again, as in all prior studies, no correlations were observed between ingroup loyalty, authority, and purity values and prosocial resource distribution. In sum, across Studies 2, 3, and 4 (Table 2: 2a, 2b, 2c), greater likelihood of prosocial choices in the social values orientation task was associated with higher valuation of caring but not ingroup loyalty, deference to authority, and purity. Coupled with the links between authority values and Machiavellianism and Social Dominance Orientation, these results suggest that in the current context, caring values were uniquely associated with a cooperative, prosocial orientation toward interactions.

These results are further refined by the results of the helping task, in which participants rated the likelihood of their own helping behaviors in scenarios involving kin/close friends and acquaintances (reported in Table 4). First, a 2 (kin/close friends, acquaintances) ×2 (help, not help) ANOVA revealed a main effect of helping indicating that participants were overall more likely to say they would help versus not help (*F*(1,116) = 489.85, *p*<.001) and a significant interaction whereby participants reported higher likelihood of *not* helping if the target was an acquaintance rather than kin or a close friend, and higher likelihood of helping if the target was kin or a close friend versus an acquaintance (*F*(1,116) = 48.90, *p*<.001). In both zero-order and partial correlations, endorsing all moral values *except* authority values was positively associated with likelihood of helping acquaintances (*p*'s<.05). In addition, the likelihood of *not* helping *both* kin/close friends and acquaintances was lower in participants higher in caring and fairness values (*p*'s<.05) again suggesting these values are related to prosociality more broadly. By contrast, this effect did not obtain for ingroup loyalty, authority, or purity values (i.e., binding values). Our initial hypothesis was that helping kin and close friends would be linked to binding values, due to their emphasis on existing partnerships and personal ties [12], [33], and that helping more distant others would be linked to individualizing values. By contrast we found that caring, fairness, ingroup loyalty and purity values correlated with reported likelihood of helping acquaintances, while only caring and fairness values were related to helping kin/close friends as well.

In sum, positive associations were observed between helping acquaintances and caring, fairness, ingroup loyalty, and purity values, *but not* authority values. In addition, higher caring and fairness values predicted *reduced* likelihood of *not* helping kin/close friends, or not helping acquaintances (caring only). Overall, these findings highlight a critical relationship between caring values and everyday prosociality, and also suggest questionable links between authority values and prosocial behavior.

## Study 5: Replication of Machiavellianism and Moral Values Findings

### Study 5 Method

Participants for Study 5 were 187 individuals who also completed unrelated measures online via Amazon.com's Mechanical Turk; 13 additional participants were excluded. These participants completed the Machiavellian Personality Scale [20] and the Moral Foundations Questionnaire [2], which allowed us to examine whether the relationships observed in Studies 1, 3 and 4 between caring values, authority values, and Machiavellianism would replicate. We conducted the same correlational analyses used in Studies 1, 3 and 4 on the Machiavellianism and MFQ data collected in Study 5 (reported in Table 1: 1d).

### Study 5 Results and Discussion

#### Moral Values and Machiavellianism

As shown in Table 1 (1d), zero-order correlational analysis showed that Mach Total score was negatively associated with caring values (*r* = −.194, *p*<.001). A negative zero-order association between the Mach Amorality subscale and caring values was also observed (*p<*.001), similar to Studies 1, 3, and 4. In line with Studies 1 and 4, on the other hand, positive zero-order correlations were observed between Mach Total score and authority values (*p<.*05) and ingroup loyalty *(p<.*05); Mach Status-Seeking and authority values (*p*<.001), ingroup loyalty (*p<.*001), and purity values (*p*<.05); Mach Control and ingroup loyalty (*p<*.05); and Mach Distrust and authority values (*p*<.05). Partial correlations controlling for religiosity, politics and gender showed that caring values remained negatively associated with Machiavellianism (Amorality: *p*<.01); and significant positive associations were retained between authority, ingroup and purity values and Mach Total, Distrust, Control, and Status-Seeking (*p*'s<.05). Thus, Study 5 replicated the findings from Studies 1, 3, and 4 and demonstrates that Machiavellianism is reliably *positively* linked with authority values and *negatively* linked with caring values.

### Meta-analysis: Machiavellianism, Prosociality, and Moral Values

**Figure 1:**
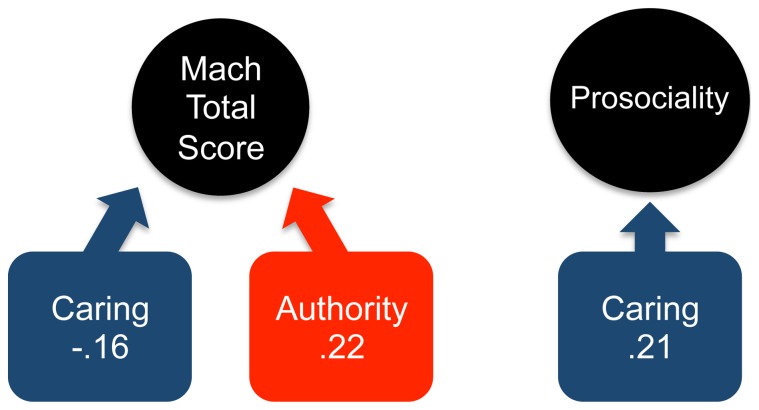
Results of Meta-Analyses. *Left*: Illustration of results of meta-analyses of data from Studies 1, 3, 4, 5 indicating a negative relationship between Caring values and Mach Total Score, and a positive relationship between Authority values and Mach Total Score. *Right*: Illustration of results of meta-analysis of data from Studies 2, 3, 4 indicating a positive relationship between Prosociality and Caring values.

While positive associations were repeatedly observed across studies between Machiavellianism and authority values, there was one study (Study 3) in which this correlation was not significant. A meta-analysis was therefore conducted to determine an aggregated correlation coefficient for the relationship between the total Machiavellianism score and authority values, with politics, religion and gender controlled. The *r* values from Study 1, 3, 4, and 5 were converted into Fisher's Z*r* effect size scores for meta-analysis. These were summed and divided by the sum of the inverse variance weights for each study (*n*-3) [34]. The resulting mean effect size was converted back into an aggregate *r* value: *r* =  .22, which indicates a small-medium effect size for the positive relationship between Machiavellianism and authority values, with politics, religion and gender controlled (Figure 1, left). In contrast, caring values were negatively associated with Machiavellianism across all studies. Nevertheless, we conducted a meta-analysis using the same procedures to determine an aggregated correlation coefficient for the relationship between the total Machiavellianism score and caring values, with politics, religion and gender controlled. Here, the resulting *r* value  =  −.16 indicates a small effect size for the negative relationship between caring values and Machiavellianism, with politics, religion and gender controlled (Figure 1, left).

In addition, caring values were also the most reliably correlated with prosociality, as measured by the social values orientation task [28]. Using the procedures above, a meta-analysis was conducted on the zero-order correlation coefficients from Studies 2, 3, and 4. The resulting *r* value  =  .21 indicates a small-medium effect size for the positive correlation between caring values and prosociality (Figure 1, right).

## General Discussion

**Figure 2:**
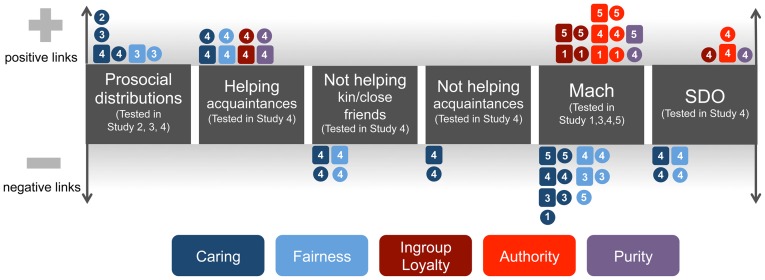
Summary of correlations observed across all studies. Each *square* represents an observation of a significant partial correlation (politics, religion, and gender controlled). Each *circle* represents an observation of a significant zero-order correlation. Study (#) indicated on each circle/square. Moral values are color-coded.

The current findings across five studies provide important insight into key moral values, in particular, values concerned with preventing harm and ensuring care, as well as values focused on respect and deference to authority (see Figure 2 and Table 5 for summaries of results across the studies). The findings reveal that authority values are not related to prosociality as measured by tasks targeting the likelihood of everyday prosocial behavior serving to build and maintain relationships with close and distant others. Instead, authority values were found to be associated with Machiavellianism and Social Dominance Orientation – manipulative interpersonal styles that keep social boundaries and hierarchical structures in place based on an “inferior/superior” continuum [21]. By contrast, prosocial resource distribution was associated with endorsement of caring values, and participants who endorsed caring values to a greater extent were less likely to report that they would deny requests for help from either close or distant others. Finally, negative associations between caring values and Machiavellianism and Social Dominance Orientation were revealed, again highlighting the potential broad connections between caring values and a generally prosocial interpersonal orientation (see Figure 2, Table 5).

**Table 5:** Summary of positive and negative correlations between moral values and prosocial and antisocial variables across all studies.

	Caring	Fairness	Ingroup	Authority	Purity
**Prosociality**	(+) Study 4, partial	(+) Study 3, partial			
	(+) Study 4	(+) Study 3			
	(+) Study 3				
	(+) Study 2				
**Helping acquaint.**	(+) Study 4, partial	(+) Study 4, partial	(+) Study 4, partial		(+) Study 4, partial
	(+) Study 4	(+) Study 4	(+) Study 4		(+) Study 4
**Not helping kin**	(−) Study 4, partial	(−) Study 4, partial			
	(−) Study 4	(−) Study 4			
**Not helping acquaint.**	(−) Study 4, partial				
	(−) Study 4				
**Machiavellianism**	(−) Study 1	(−) Study 3, partial	(+) Study 1, partial	(+) Study 1, partial	(+) Study 4
	(−) Study 3, partial	(−) Study 3	(+) Study 1	(+) Study 1	(+) Study 5, partial
	(−) Study 3	(−) Study 4, partial	(+) Study 5, partial	(+) Study 3, partial	
	(−) Study 4, partial	(−) Study 4	(+) Study 5	(+) Study 3	
	(−) Study 4	(−) Study 5		(+) Study 5, partial	
	(−) Study 5, partial			(+) Study 5	
	(−) Study 5				
**SDO**	(−) Study 4, partial	(−) Study 4, partial	(+) Study 4	(+) Study 4, partial	(+) Study 4
	(−) Study 4	(−) Study 4		(+) Study 4	

**Notes**. “Partial” refers to partial correlations with political orientation, religiosity, and gender controlled; (+) indicates significant positive correlation, (−) indicates significant negative correlation.

What could account for the positive relationship between respect for authority and Machiavellianism, an antisocial interpersonal style associated with strategic manipulation? Indeed, Machs have been shown to lie more convincingly [35], steal more readily [36], and rationalize deeds with callous unemotionality [37]. To provide the foundation for two potential explanations for this surprising relationship, we first describe two relevant aspects of Machiavellianism: (1) Machiavellianism and psychopathy are distinct in relation to social norm processing, and (2) Machs are likely to be dominant individuals in positions of authority. Next, we propose two potential explanations for the positive relationships between moral valuation of respect for authority and Machiavellianism: (1) Machiavellianism may entail moralization of respect for authority for a variety of strategic reasons, and (2) authority values may license Machiavellian behavior. Finally, after discussing the links between moral values and prosociality, we note important caveats regarding the correlational nature of these studies, and highlight several rich areas for future research.

### Distinctions between Machiavellianism and Psychopathy

Although Machiavellianism is characterized by selfishness and shares some overlap with psychopathy [23], Machs are not necessarily aloof and unconcerned with social norms. Instead, the ability to manipulate others may actually benefit from a keen sensitivity to norms that govern social structure. Supporting this specific link, aloofness has been found to be negatively associated with the manipulative/deceptive hierarchy negotiation tactics that theoretically align with Machiavellianism [38]. Moreover, recent experimental work has shown that high Machs achieved greater profit than low Machs in a public goods game by strategically and continuously monitoring their opponent and adjusting their own moves accordingly [39]. Likewise, individuals higher in Machiavellianism earned more money in an economic game that involved distributing resources to the self and another under two conditions: threat of retaliation or not [40]. Here, Machs gave the least and conserved the most when they could not be punished, but they escaped punishment by substantially increasing their giving when punishment was possible. Moreover, brain activation patterns suggested that Machs were especially sensitive to the punishing stimuli, which contrasts with activation patterns shown by psychopathic individuals who disregard punishment signals [40], [41]. Thus, while Machiavellianism and psychopathy overlap to some degree [23], these personality types may nevertheless be conceptually distinct in their relation to moral values. Machs' heightened attunement to social signals likely allows them to perceive the potential self-serving benefits of moralizing respect for authority and social structures [42]–[45]. We detail these potential moralization processes in the section “Machiavellian Moralization” below.

### Machiavellianism and Dominance

In addition to being hyper-attuned to social structure, Machs are also likely to reside at the top of those structures in positions of authority. Machiavellian-style social climbing tactics (e.g., manipulation and deception) are more likely to be used by individuals high in dominance and well-equipped to assume authority over others [38]. Likewise, Machiavellian supervisors in a range of business sectors have been described by subordinates as employing authoritarian work habits involving strict control over a hierarchical workplace structure [46]. As individuals who recognize they can personally benefit from “working the system” from a position of authority – rather than attempting to make the system work for all – Machs may be more likely to identify respect for authority as relevant or even central to their concepts of “right and wrong”.

### Machiavellian Moralization

Ascribing moral relevance to a preference typically implies an extension of judgment on the topic from the self to the other – if something is right or wrong for me, it is also right or wrong for you [43], [47]–[50]. Since Machs are likely to be in influential, dominant positions, Machs may be more likely to promote authority valuation among their subordinates. Proselytizing their moralization of respect for authority would then help Machs maintain acquiescence from those they dominate since adherence to these values will, by their nature, help keep hierarchical structures intact. Thus, a strategic benefit of moralization of respect for authority may be that it helps Machs maintain a dominant position through promotion of this moral position to subordinates.

In addition to altering the behavior of their subordinates, moralization of respect for authority might also help Machs regulate their own behavior to meet self-interested motives. Besides being powerfully emotionally comforting [51], deference to the authorities within one's own group may serve to guarantee one's own protection by the group, as has been observed for those ingroup members who are more engaged in system justification [52]. Adopting a moral stance related to authority may be a way for Machs to project the appearance of a moral leader [42], [53].

Finally, further utility for Machs may be found in the facilitative effects of moralization, as behaviors and preferences given moral relevance become more automatic [43]–[45]. Thus, moralization of respect for authority may make the self-serving Machiavellian tactics of flattery and ingratiation to superiors easier to maintain [54].

### Machiavellian Outcomes

Alternatively, authority values may themselves facilitate Machiavellian-like behavior, particularly behaviors described in the Mach Amorality subscale, because these values prescribe deference to authority over unconditional respect for individuals' basic human rights and dignity. For example, when situations arise that pit these concerns against each other, respect for authority, tradition, and extant social structures may be upheld even in the case of what might otherwise be considered morally “bad” behavior (e.g., bribing, cheating, hazing, or torture). Moreover, recent work suggests that individuals primed to feel high in power – that is, closer to “authority figure” status – were more likely to endorse unethical and antisocial behavior [55], [56]. Notably, the approach-orientation of the powerful leads them to focus more on what they should be doing (good outcomes), rather than what they should not be doing (bad outcomes), which has the effect of licensing morally wrong behavior [56]. This work broadens the self-reinforcing link between authority values in general and Machiavellian-like behavior. Simply seeking to attain a powerful position may induce unethical behavior and grant one a personal stake in whether authority values deserve moral status.

### Moral Values and Links with Prosociality

Negative associations between caring values and Machiavellianism (Mach Amorality, Control, and Status-Seeking), Social Dominance Orientation, and failing to help both close and distant others, coupled with positive links between caring values and prosocial resource distribution broadly underscore the potential link between these values and everyday prosocial behavior.

In the social values orientation task, prosocial choices maximized good outcomes by equalizing resources between one's self and another person (rather than maximizing good outcomes for one's self at the expense of another person) [28]. Thus, it appears that endorsement of moral values that prioritize caring, preventing harm, and protecting others was associated with the kind of fair and unselfish resource distributions that most people would appreciate in everyday life. In addition, when rating the likelihood of helpful behaviors based on scenarios involving kin/close friends and acquaintances, caring values were associated with reduced likelihood of denying requests for help. This suggests that caring values are related to prosociality broadly – both in close relationships and across group boundaries.

Authority values have been bundled with ingroup loyalty and purity values in theoretical arguments that collectively frame them as the “binding values” [1], [2], [8], [32]. In several places, we found authority values to track with antisocial variables, whereas ingroup values and purity values either did not, or did so inconsistently. For example, although the binding values of ingroup loyalty and purity were associated with helping more distant others along with caring and fairness values, authority values were not; and, of all the binding values, only authority values remained positively correlated with SDO when politics, religiosity and gender were controlled. These findings reveal important dissociations within the “binding values” and, once again, the potential for a dark side of authority values. Authority values do not specifically prohibit harming others and instead relate to maintaining social boundaries; thus, selfish or non-cooperative behavior (as in the current study) may be wholly in keeping with these values [57]. Meanwhile, our observation of a positive correlation between ingroup loyalty valuation and helping acquaintances may be less surprising since help requesters in this condition were people who, while not family or friends, were still described as living or working nearby. Thus, coalition formation/maintenance, related to ingroup loyalty, may be a likely motivator in such interactions. Future work should explore other ways in which ingroup loyalty and purity values diverge from authority values.

### Limitations and Future Directions

These studies provide evidence of correlations between moral values on the one hand and prosocial and antisocial interpersonal orientations on the other hand. We note that the correlational results do not allow us to discern the causal nature of these links. It may be that unmeasured variables account for these relationships. For example, Machiavellian individuals have been shown to be anxious [58] and hyper-attuned to punishing stimuli [40]. Likewise, anxious individuals who find punishment especially salient may be those who are most familiar and comfortable in authoritarian environments that foster authority values, making punitive upbringing a potential “third variable” here.

Furthermore, the direction of the observed connections cannot yet be determined. While we have provided possible reasons for these links in both directions (i.e., how moral values could lead to interpersonal orientations, and how these orientations could lead to elevation of certain moral values), it may also be the case that authority values, linked here with antisocial tendencies, also track with other, unmeasured positive outcome variables. Future work using experimental methods, and not correlational designs, will be necessary to investigate whether Machiavellianism and authority values are causally connected and the direction of causality. For example, if Machs are motivated to moralize respect for authority, then endorsement of authority values should increase when individuals are primed to behave in a Machiavellian manner, e.g., when Machiavellian concerns (e.g., trust, status, control, strategy) are made relevant to goal pursuit. Alternatively, it may the case that manipulative and deceptive tendencies increase when authority values are primed.

Incidentally, recent research has also revealed positive links between sexism and binding values, and negative links between sexism and individualizing values [59]. If sexist attitudes, like Machiavellianism, are assumed to represent an antisocial and undesirable interpersonal orientation involving a desire for dominance and manipulation, these findings together underscore the need for investigation into motivated moralization as an explanation for these striking associations.

Finally, future work should aim to determine whether these links play out on a larger scale in group cultures. Should we expect to find more Machiavellian individuals in institutions or organizations that emphasize authority values? Are prosocial individuals more plentiful in settings that codify universal caring? Whether these individual-level correlations extend to patterns at the organizational level is another fruitful area for exploration.

### Implications

#### Links with Behavior

The importance of correlations between authority values and Machiavellianism is underscored by research demonstrating that Machs not only perform more unethical behaviors (e.g., cheating, stealing, sabotage) but may also carry psychological burdens from these behaviors including anxiety, low job and life satisfaction, and feelings of disconnection [58], [60].

By contrast, the prosociality measure (SVO) [28] related to caring values has previously been linked with behaviors that underlie positive social interactions, some of which may be unexpected. For example, a tendency to show embarrassment – a subjectively unpleasant experience that actually serves to signal legitimate good will and establish trust – is linked to prosocial choices [30]. Also, those ranking higher in prosociality on this task were found to be more likely to negatively evaluate procedures in a task when people other than themselves were denied a voice in the procedure even though they were themselves granted a voice; this result suggests these individuals not only care about fair resource distribution but also may be willing to advocate on behalf of those denied justice [61]. Thus, the independent constructs we found to relate to moral values may have real importance for social functioning at large.

#### Meta-ethical Implications

Questions about the roots of morality emerge at the descriptive level [42], [62]–[64] as well as the meta-ethical level – what we *ought* to count as morality. To the extent that lying and cheating and otherwise manipulative strategies reflect morally bad behavior, whereas prosocial resource distribution and meeting others' requests for help reflect morally good behavior, the present findings highlight the overarching importance of caring values and raise questions about the normative status of other values – specifically, authority values.

Of course, it might also be a coincidence that authority values track with antisocial tendencies. As a helpful anonymous reviewer pointed out, suppose we had discovered that people with antisocial tendencies endorsed not authority values but a certain kind of music – should that change our normative view of that music? Most likely not. However, unlike the pairing of music preferences and antisocial tendencies, authority values and antisocial tendencies are both of moral relevance; therefore, a closer analogue might be the discovery of a renowned musical genius' endorsement of a certain kind of music. Should this endorsement affect our attitude toward that music? Perhaps so.

Disagreements over moral values are often unavoidable because many of us hold not simply different moral values but also the view that our moral values represent factual truths [47]–[50], [65]. The present research as well as other work using this empirical approach [18], [19] can provide a unique foothold in the midst of moral diversity and point to a clearer picture of how moral values are linked to particular interpersonal orientations and everyday social outcomes.

## Supporting Information

File S1 **Includes Text, Appendixes S1 – S6 and Table S1.**
(DOC)
