# Supplementary material for: Caring across Boundaries versus Keeping Boundaries Intact: Links between Moral Values and Interpersonal Orientations
Source: PLoS One. 2013 Dec 12;8(12):e81605. doi: 10.1371/journal.pone.0081605 (PMC3861283; doi:10.1371/journal.pone.0081605)
Supplement: File S1 — Includes Text, Appendixes S1 – S6 and Table S1. (DOC) [file pone.0081605.s001.doc]

**Supporting** **Information File S1**

**Supporting Text A.** We describe our exploratory investigation of the contributions of guilt and shame to associations between moral values and Machiavellianism in Study 3.

**Table S1.** We report partial correlations between moral Values and Machiavellianism with guilt and shame dimensions controlled: guilt-related negative behavior evaluations (Guilt NBE; Table S1a), guilt-related repair tendencies (Guilt Repair; Table S1b), shame-related negative self evaluations (Shame NSE; Table S1c), and shame-related withdrawal tendencies (Shame Withdrawal; Table S1d).

**Appendix S1.** 30-item Moral Foundations Questionnaire.

**Appendix S2.** Machiavellian Personality Scale.

**Appendix S3.** Helping Task items.

**Appendix S4.** Social Dominance Orientation scale.

**Appendix S5.** Guilt and Shame Proneness scale.

**Appendix S6.** Here we provide a series of tables containing the results of all analyses presented in the main text conducted without any exclusions. For ease of comparison, the results for the samples without any exclusions are italicized directly over the main results.

***A. Exploring the Role of Guilt and Shame in Associations between Moral Values and Machiavellianism (Study 3)***

In exploratory analyses, we investigated the contributions of guilt and shame to associations between moral values and Machivellianism. On the one hand, negative behavior evaluations and reparative behaviors motivated by guilt as well as negative self-evaluations motivated by shame have been linked to ethical decision-making and endorsement of a range of moral values [1-3]. On the other hand, maladaptive shame regulation – behavioral withdrawal after experiencing feelings of shame – has been hypothesized to predict Machiavellianism [4] and has also been linked to unethical and antisocial behavior [2]. Therefore, we were interested in whether guilt and shame factored into relationships between Machiavellianism and moral values in Study 3.

*Method*

In Study 3, participants’ guilt-related negative behavior evaluations (“Guilt NBE”), shame-related self-evaluations (“Shame NSE”), likelihood of repair behavior in response to guilt (“Guilt-Repair”), and withdrawal behavior in response to shame (“Shame-Withdrawal”) were assessed using the Guilt and Shame Proneness Scale (GASP) [2]; see Appendix S5 for items). For all participants in Study 3, the GASP was presented after the dependent measures reported in the main text. In Study 1, we also ran two of the GASP subscales (“Shame-Withdrawal”, “Guilt-Repair”) and other newly constructed items probing guilt and shame; for all participants, these items were presented after the dependent measures reported in the main text. Only the exploratory analyses of data from Study 3 are reported here.

We report partial correlations between the moral values (measured with the MFQ) [5]; and Machiavellianism (measured with the MPS) [6]; controlling for each of the four guilt and shame subscale scores separately (Table S1: a, b, c, d).

*Results*

We tested the relationships between the moral values and Machiavellianism in a series of partial correlational analyses, separately controlling for each of the four guilt and shame subscales. As reported in Table S1: a, b, c, controlling for the three aspects of guilt and shame that have been previously linked with ethical decision-making – guilt-related negative behavior evaluations and reparative behaviors (Guilt NBE: Table S1: a & Guilt-Repair: Table S1: b) and shame-related negative self-evaluations (Shame NSE: Table S1: c) – changed the strength of the associations reported for Study 3 in the main text in two ways. First, in all three of these sets of analyses, the positive associations between Machiavellianism and authority values increased from non-significant trends (see Table 1: 1b in the main text) to *significant positive correlations* – aligning closely with the key results of Studies 1 and 4 reported in the main text. In addition, ingroup loyalty and purity values were also positively associated with Machiavellianism. Second, negative associations found in Study 3 (see Table 1 :1b in the main text) between Machiavellianism and caring values were reduced to non-significance in these three sets of analyses (Tables S1: a, b, c). However, a fourth set of partial correlational analyses controlling for the maladaptive shame response (Shame-Withdrawal; Table S1: d) revealed the same significant negative links between caring values and Machiavellianism, and no associations between authority, ingroup loyalty, and purity values and Machiavellianism, as found in the main text (see Table 1: 1b in the main text).

Taken together (Table S1: a, b, c, d), these exploratory analyses indicate that guilt and shame and related behaviors, as measured using the GASP, can alter the strength of associations between moral values and Machiavellianism. Nevertheless, the overall pattern of the observed associations remains consistent: *negative* links between caring values (and also fairness in some cases) and Machiavellianism, and *positive* links between authority values (and also ingroup loyalty and purity values in some cases) and Machiavellianism. Future research should explore how moral values in different domains (e.g., caring versus authority values) function alongside emotional and behavioral tendencies related to guilt and shame to predict the kind of unethical decision-making that would be expected from Machs.

|  | **Table S1.**  *Moral Values & Machiavellianism with Guilt NBE, Guilt Repair, Shame NSE, Shame Withdrawal Controlled: Partial Correlations from Study 3.* | | | | | | |
| --- | --- | --- | --- | --- | --- | --- | --- |
|  |  | **Mach Total** | **Mach Amoral.** | **Mach Control** | **Mach Status** | **Mach Distrust** | |
| **S1a.**  **Guilt NBE Controlled** | CARING | -.100  *p=*.289 | -.090  *p=*.340 | -.150  *p=*.110 | -.079  *p=*.405 | -.010  *p=*.912 | |
|  | FAIRNESS | -.008  *p=*.934 | -.066  *p=*.482 | **-.227^*^**  *p=*.015 | -.048  *p=*.609 | .040  *p=*.671 | |
|  | INGROUP | **.352^***^**  *p=*.000 | **.298^***^**  *p=*.001 | **.289^**^**  *p=*.002 | **.313^***^**  *p=*.001 | **.**182  *p=*.052 | |
|  | AUTHORITY | **.296^***^**  *p=*.001 | **.203^*^**  *p=*.030 | **.207^*^**  *p=*.027 | **.310^***^**  *p=*.001 | **.**176  *p=*.061 | |
|  | PURITY | **.303^***^**  *p=*.001 | **.239^**^**  *p=*.010 | .142  *p=*.133 | **.293^**^**  *p=*.002 | **.221^*^**  *p=*.018 | |
| **S1b.**  **Guilt Repair Controlled** | CARING | -.123  *p=*.193 | -.134  *p=*.155 | -.086  *p=*.365 | -.119  *p=*.207 | -.045  *p=*.636 | |
|  | FAIRNESS | .033  *p=*.725 | .091  *p=*.334 | -.146  *p=*.122 | .053  *p=*.578 | .053  *p=*.576 | |
|  | INGROUP | **.232^*^**  *p=*.013 | .141  *p=*.134 | **.288^**^**  *p=*.002 | **.226^*^**  *p=*.016 | **.**102  *p=*.279 | |
|  | AUTHORITY | **.261^**^**  *p=*.005 | .147  *p=*.119 | **.258^*^**  *p=*.006 | **.277^**^**  *p=*.003 | **.**149  *p=*.113 | |
|  | PURITY | **.207^*^**  *p=*.027 | .115  *p=*.222 | .146  *p=*.121 | **.222^*^**  *p=*.017 | .154  *p=*.102 | |
| **S1c.**  **Shame NSE controlled** | CARING | -.169  *p=*.072 | -.176  *p=*.061 | -.147  *p=*.120 | -.161  *p=*.087 | -.053  *p=*.573 | |
|  | FAIRNESS | -.072  *p=*.449 | -.022  *p=*.817 | **-.226^*^**  *p=*.015 | -.025  *p=*.791 | .002  *p=*.985 | |
|  | INGROUP | **.197^*^**  *p=*.035 | .112  *p=*.234 | **.241^**^**  *p=*.010 | **.193^*^**  *p=*.039 | **.**194  *p=*.321 | |
|  | AUTHORITY | **.218^**^**  *p=*.020 | .111  *p=*.239 | **.203^*^**  *p=*.030 | **.235^*^**  *p=*.012 | **.**138  *p=*.143 | |
|  | PURITY | .161  *p=*.088 | .071  *p=*.455 | .098  *p=*.298 | **.185^*^**  *p=*.049 | .134  *p=*.154 | |
| **S1d.**  **Shame Withdrawal controlled** | CARING | **-.339^***^**  *p=*.000 | **-.356^***^**  *p=*.000 | **-.254^**^**  *p=*.006 | **-.240^***^**  *p=*.010 | **-.190^*^**  *p=*.043 | |
|  | FAIRNESS | **-.218^*^**  *p=*.020 | **-.189^*^**  *p=*.044 | **-.309^***^**  *p=*.001 | -.094  *p=*.317 | -.112  *p=*.235 | |
|  | INGROUP | **.**014  *p=*.882 | **-.**062  *p=*.511 | **.**125  *p=*.185 | **.**083  *p=*.379 | **-.**051  *p=*.593 | |
|  | AUTHORITY | **.**020  *p=*.832 | **-.**081  *p=*.389 | **.**077  *p=*.416 | **.**122  *p=*.195 | **-.**015  *p=*.873 | |
|  | PURITY | **.**003  *p=*.978 | **-.**073  *p=*.442 | **.**001  *p=*.988 | **.**087  *p=*.360 | **.**008  *p=*.929 | |
| *Notes.* NBE = negative behavior evaluations, NSE = negative self-evaluations. * *p*<.05, ***p*<.01, ****p*<.001 | | | | | | |  |

**Appendix S1.** 30-item Moral Foundations Questionnaire (Likert-Scale Scored from 1-6) (with two catch questions (6 and 22) [5]

*When you decide whether something is right or wrong, to what extent are the following*

*considerations relevant to your thinking? (Not at all relevant to Extremely relevant)*

1. Whether or not someone suffered emotionally ***(Caring)***

2. Whether or not some people were treated differently than others ***(Fairness)***

3. Whether or not someone’s action showed love for his or her country ***(Ingroup Loyalty)***

4. Whether or not someone showed a lack of respect for authority ***(Authority)***

5. Whether or not someone violated standards of purity and decency ***(Purity)***

*6. Whether or not someone was good at math (attention check)*

7. Whether or not someone cared for someone weak or vulnerable ***(Caring)***

8. Whether or not someone acted unfairly ***(Fairness)***

9. Whether or not someone did something to betray his or her group ***(Ingroup Loyalty)***

10. Whether or not someone conformed to the traditions of society ***(Authority)***

11. Whether or not someone did something disgusting ***(Purity)***

12. Whether or not someone was cruel ***(Caring)***

13. Whether or not someone was denied his or her rights ***(Fairness)***

14. Whether or not someone showed a lack of loyalty ***(Ingroup Loyalty)***

15. Whether or not an action caused chaos or disorder ***(Authority)***

16. Whether or not someone acted in a way that God would approve of ***(Purity)***

*Please read the following sentences and indicate your agreement or disagreement:*

17. Compassion for those who are suffering is the most crucial virtue. ***(Caring)***

18. When the government makes laws, the number one principle should be ensuring that everyone is treated fairly. ***(Fairness)***

19. I am proud of my country’s history. ***(Ingroup Loyalty)***

20. Respect for authority is something all children need to learn. ***(Authority)***

21. People should not do things that are disgusting, even if no one is harmed. ***(Purity)***

*22. It is better to do good than to do bad. (attention check)*

23. One of the worst things a person could do is hurt a defenseless animal. ***(Caring)***

24. Justice is the most important requirement for a society. ***(Fairness)***

25. People should be loyal to their family members, even when they have done something wrong. ***(Ingroup Loyalty)***

26. Men and women each have different roles to play in society. ***(Authority)***

27. I would call some acts wrong on the grounds that they are unnatural. ***(Purity)***

28. It can never be right to kill a human being. ***(Caring)***

29. I think it’s morally wrong that rich children inherit a lot of money while poor children inherit nothing. ***(Fairness)***

30. It is more important to be a team player than to express oneself. ***(Ingroup Loyalty)***

31. If I were a soldier and disagreed with my commanding officer’s orders, I would obey anyway because that is my duty. ***(Authority)***

32. Chastity is an important and valuable virtue. ***(Purity)***

*Note.* Failure on the attention check questions was defined as answering (5) or (6) to Question 6, or (1) or (2) to Question 22.

**Appendix S2.** Machiavellian Personality Scale (Likert-Scale Scored from 1-5: Completely Disagree to Completely Agree) [6]

***Amorality Subscale***

- I believe that lying is necessary to maintain a competitive advantage over others.
- The only good reason to talk to others is to get information that I can use to my benefit.
- I am willing to be unethical if I believe it will help me succeed.
- I am willing to sabotage the efforts of other people if they threaten my own goals.
- I would cheat if there was a low chance of getting caught.

***Desire for Control Subscale***

- I like to give the orders in interpersonal situations.
- I enjoy having control over other people.
- I enjoy being able to control the situation.

***Desire for Status Subscale***

- Status is a good sign of success in life.
- Accumulating wealth is an important goal for me.
- I want to be rich and powerful someday.

***Distrust of Others Subscale***

- People are only motivated by personal gain.
- I dislike committing to groups because I don’t trust others.
- Team members backstab each other all the time to get ahead.
- If I show any weakness at work, other people will take advantage of it.
- Other people are always planning ways to take advantage of the situation at my expense.

**Appendix S3.** Helping Task Items (Participants received one of each of the four types of items; Likert-Scale Scored from 1-7: Not at all likely to Very likely)

*What is the likelihood that you would have done the same thing as [PROTAGONIST]?*

***Helping Close Other***

- Caroline’s mother asks her if she would pick up some forms for her from an office downtown on Tuesday. Tuesday comes around and Caroline picks up the forms.
- Jeff’s brother asks him if he will help move some branches out of his yard next week. The following week, Jeff helps move the branches.
- Kenneth’s best friend asks Kenneth if he could drop him off at the airport on his way to work Thursday evening. On Thursday evening, Kenneth brings him to the airport.
- Lisa’s best friend asks Lisa if she will let her store a bureau in her basement for a couple months. Lisa decides to let her store the bureau in her basement.

***Not Helping Close Other***

- Caroline’s mother asks her if she would pick up some forms for her from an office downtown on Tuesday. Tuesday comes around and Caroline doesn’t pick up the forms.
- Jeff’s brother asks him if he will help move some branches out of his yard next week. The following week, Jeff doesn't help move the branches.
- Kenneth’s best friend asks Kenneth if he could drop him off at the airport on his way to work Thursday evening. On Thursday evening, Kenneth doesn’t bring him to the airport.
- Lisa’s best friend asks Lisa if she will let her store a bureau in her basement for a couple months. Lisa decides to not let her store the bureau in her basement.

***Helping Distant Other***

- Caroline’s neighbor down the street asks her if she would pick up some forms for her from an office downtown on Tuesday. Tuesday comes around and Caroline picks up the forms.
- A man who works next door to Jeff asks if he will help move some branches out of his yard next week. The following week, Jeff helps move the branches.
- A neighbor who just moved in nearby asks Kenneth if he could drop him off at the airport on his way to work Thursday evening. On Thursday evening, Kenneth brings him to the airport.
- A woman who lives nearby asks Lisa if she will let her store a bureau in her basement for a couple months. Lisa decides to let her store the bureau in her basement.

***Not Helping Distant Other***

- Caroline’s neighbor down the street asks her if she would pick up some forms for her from an office downtown on Tuesday. Tuesday comes around and Caroline doesn’t pick up the forms.
- A man who works next door to Jeff asks if he will help move some branches out of his yard next week. The following week, Jeff doesn’t help move the branches.
- A neighbor who just moved in nearby asks Kenneth if he could drop him off at the airport on his way to work Thursday evening. On Thursday evening, Kenneth doesn’t bring him to the airport.
- A woman who lives nearby asks Lisa if she will let her store a bureau in her basement for a couple months. Lisa decides to not let her store the bureau in her basement.

**Appendix S4.** Social Dominance Orientation scale (Participants received one of each item; Likert-Scale Scored from 1-7: Very negative to Very positive) [7]

*Do you have a positive or negative feeling towards the following statements?*

*Beside each statement, rate how you feel about it, from 1 (very negative) to 7 (very*

*positive).*

- It's probably a good thing that certain groups are at the top and other groups are at the bottom.
- It's OK if some groups have more of a chance in life than others.
- We should do what we can to equalize conditions for different groups.
- All groups should be given an equal chance in life.
- In getting what you want, it is sometimes necessary to use force against other groups.
- If certain groups stayed in their place, we would have fewer problems.
- We would have fewer problems if we treated people more equally.
- To get ahead in life, it is sometimes necessary to step on other groups.
- Some groups of people are simply inferior to other groups.
- Group equality should be our ideal.
- We should strive to make incomes as equal as possible.
- Inferior groups should stay in their place.
- It would be good if groups could be equal.
- No one group should dominate in society.
- Sometimes other groups must be kept in their place.
- Increased social equality.

**Appendix S5.** Guilt and Shame Proneness Scale Items (Likert-Scale Scored from 1-7: Very Unlikely to Very Likely) [2]

***Guilt–Negative-Behavior-Evaluation***

1. After realizing you have received too much change at a store, you decide to keep it because the salesclerk doesn’t notice. What is the likelihood that you would feel uncomfortable about keeping the money?

9. You secretly commit a felony. What is the likelihood that you would feel remorse about breaking the law?

14. At a coworker’s housewarming party, you spill red wine on their new cream-colored carpet. You cover the stain with a chair so that nobody notices your mess. What is the likelihood that you would feel that the way you acted was pathetic?

16. You lie to people but they never find out about it. What is the likelihood that you would feel terrible about the lies you told?

***Guilt–Repair***

2. You are privately informed that you are the only one in your group that did not make the honor society because you skipped too many days of school. What is the likelihood that this would lead you to become more responsible about attending school?

5. You reveal a friend’s secret, though your friend never finds out. What is the likelihood that your failure to keep the secret would lead you to exert extra effort to keep secrets in the future?

11. You strongly defend a point of view in a discussion, and though nobody was aware of it, you realize that you were wrong. What is the likelihood that this would make you think more carefully before you speak?

15. While discussing a heated subject with friends, you suddenly realize you are shouting though nobody seems to notice. What is the likelihood that you would try to act more considerately toward your friends?

***Shame–Negative-Self-Evaluation***

3. You rip an article out of a journal in the library and take it with you. Your teacher discovers what you did and tells the librarian and your entire class. What is the likelihood that this would make you would feel like a bad person?

6. You give a bad presentation at work. Afterwards your boss tells your coworkers it was your fault that your company lost the contract. What is the likelihood that you would feel incompetent?

10. You successfully exaggerate your damages in a lawsuit. Months later, your lies are discovered and you are charged with perjury. What is the likelihood that you would think you are a despicable human being?

13. You make a mistake at work and find out a coworker is blamed for the error. Later, your coworker confronts you about your mistake. What is the likelihood that you would feel like a coward?

***Shame–Withdrawal***

4. After making a big mistake on an important project at work in which people were depending on you, your boss criticizes you in front of your coworkers. What is the likelihood that you would feign sickness and leave work?

7. A friend tells you that you boast a great deal. What is the likelihood that you would stop spending time with that friend?

8. Your home is very messy and unexpected guests knock on your door and invite themselves in. What is the likelihood that you would avoid the guests until they leave?

12. You take office supplies home for personal use and are caught by your boss. What is the likelihood that this would lead you to quit your job?

**Appendix S6.** Results of all analyses reported in the main text conducted without exclusions.

The following tables contain the results of all analyses presented in the main text conducted without any exclusions. For ease of comparison, the results for the samples without any exclusions are *italicized* directly over the main results.

***Study 1: n=132***

***Mach Mach Mach Mach Mach***

***Amorality Control Status Distrust TOTAL***

***(partial) (partial) (partial) (partial) (partial)***

***-.335*** -.278****** *-.087 -.062* ***-.243*****

*CARING* ***-.313*********-.235***** *-.022 -.035* ***-.196****

***-.237***** *-.149 -.003 -.003 -.130*

*FAIRNESS* ***-.224**** *-.120 .042 .016 -.097*

***.271***** *.118* ***.427*** .297** .359******

*INGROUP* ***.309****** *.149* ***.467*** .325*** .406******

***.215****  *.063* ***.330*** .254** .282******

*AUTHORITY* ***.295****** *.118* ***.393*** .294*** .361******

*.022 -.016 .091 .119 .073*

*PURITY .084 .060 .156 .167 .153*

**Study 1: n=117 (15 exclusions^1^)**

**-.235* -.231*** -.043 -.019 -.165

CARING -.169 -.158 .051 .041 -.070

-.164 -.112 .024 .042 -.065

FAIRNESS -.121 -.052 .100 .090 .010

**.218*** .072 **.417*** .256** .323****

INGROUP **.294**** .102 **.458*** .297*** .394*****

.113 -.018 **.293** .199* .203***

AUTHORITY **.235*** .030 **.362*** .256** .308*****

-.063 -.049 .075 .082 .019

PURITY .065 .033 .144 .158 .140

Notes. **^1^**Exclusions for all studies were made based on failing the catch questions included in the MFQ, or completing a section of 8 MFQ questions in under 10 seconds. “Partial” refers to partial correlations with political orientation, religiosity, and gender controlled. Zero-order correlation coefficient is presented on top, partial correlation coefficient is underneath. Boldface indicates significant correlations. * p<.05, **p<.01, ***p<.001

***Study 3: n=122***

***Mach Mach Mach Mach Mach***

***Amorality Control Status Distrust TOTAL***

***(partial) (partial) (partial) (partial) (partial)***

***-.387*** -.263** -.229* -.198* -.333******

*CARING* ***-.297*** -.214**** *-.177 -.139* ***-.257*****

***-.286*** -.325****** *-.120 -.126* ***-.261*****

*FAIRNESS* ***-.206*******-.293****** *-.066 -.069* ***-.193****

*.041* ***.179**** *.168 .076 .150*

*INGROUP .073 .153 .141 .085 .146*

*.002 .124 .176 .060 .120*

*AUTHORITY .045 .119 .163 .061 .129*

*.036 .071 .158 .100 .119*

*PURITY .081 .047 .137 .100 .118*

**Study 3: n= 115 (7 exclusions)**

**-.351*** -.255** -.235* -.190* -.324*****

CARING **-.234* -.188*** -.170 -.122 **-.223***

**-.189* -.306***** -.098 -.114 **-.210***

FAIRNESS -.105 **-.279**** -.051 -.058 -.141

-.013 .160 .137 .013 .079

INGROUP .028 .136 .111 .021 .084

-.039 .108 .164 .036 .074

AUTHORITY .029 .106 .153 .043 .098

-.022 -.043 .142 .069 .071

PURITY .048 .019 .124 .073 .087

Notes. “Partial” refers to partial correlations with political orientation, religiosity, and gender controlled. Zero-order correlation coefficient is presented on top, partial correlation coefficient is underneath. Boldface indicates significant correlations. * p<.05, **p<.01, ***p<.001

***Study 4: n= 129***

***Mach Mach Mach Mach Mach***

***Amorality Control Status Distrust TOTAL***

***(partial) (partial) (partial) (partial) (partial)***

***-.242** -.260** -.335****** *-.041* ***-.310******

*CARING* ***-.245** -.222* -.316****** *-.062* ***-.298**************

*-.160 -.144 -.149* ***-****.003 -.158*

*FAIRNESS* ***-.192**** *-.112 -.127* ***-****.003 -.149*

*-.083 -.049 .086 .019 .000*

*INGROUP -.017 -.056 .100 -.013 .014*

*.059 .017* ***.229***** *.069 .141*

*AUTHORITY* ***.****153**.020* ***.267***** *.042* ***.178****

*-.122 -.051 .088* ***.187**** *.041*

*PURITY -.016 -.086 .097 .136 .052*

**Study 4: n= 117 (12 exclusions)**

**-.216* -.263** -.390***** -.011 **-.279****

CARING **-.215* -.238* -.381***** -.035 **-.277********

-.121 -.137 **-.193*** .012 -.138

FAIRNESS -.167 -.120 **-.188*** .011 -.148

-.117 -.040 .043 .029 -.029

INGROUP -.037 -.044 .058 .007 -.003

.071 .058 **.202*** .120 .156

AUTHORITY **.207*** .060 **.241**** .102 **.213***

-.149 -.033 .051 **.212*** .038

PURITY .004 -.066 .064 .169 .076

Notes. “Partial” refers to partial correlations with political orientation, religiosity, and gender controlled. Zero-order correlation coefficient is presented on top, partial correlation coefficient is underneath. Boldface indicates significant correlations. * p<.05, **p<.01, ***p<.001

***Study 5: n=200***

***Mach Mach Mach Mach Mach***

***Amorality Control Status Distrust TOTAL***

***(partial) (partial) (partial) (partial) (partial)***

***-.333****** *-.049 -.134* ***-.177* -.252*****

*CARING* ***-.278****** *-.012 -.059 -.105* ***-.169****

***-.157* -.****037 -.110 -.065 -.129*

*FAIRNESS -.128 -.018 -.064 .032 -.058*

*-.090* ***.175* .314****** *.106* ***.147****

*INGROUP -.011* ***.191** .338****** *.127* ***.200*****

*-.087 .072* ***.275*** .171* .139****

*AUTHORITY .013 .085* ***.313*** .199** .205*****

***-.186***** *.038* ***.139**** *.075 .012*

*PURITY -.032 .094* ***.232*** .147* .145****

**Study 5: n=187 (13 exclusions)**

**-.287***** -.007 -.103 -.129  **-.194****

CARING **-.219**** .041 -.017 -.054 -.097

-.022 **.**031 -.060 .020 -.011

FAIRNESS .011 .052 -.014 .106 .059

-.115 **.185* .328***** .090 **.146***

INGROUP -.031 **.197** .347***** .115 **.199****

-.076 .093 **.313*** .187* .174***

AUTHORITY .044 .106 **.352*** .228** .253*****

**-.196**** .053 **.155*** .178 .023

PURITY -.027 .108 **.244*** .172* .169***

Notes. “Partial” refers to partial correlations with political orientation, religiosity, and gender controlled. Zero-order correlation coefficient is presented on top, partial correlation coefficient is underneath. Boldface indicates significant correlations. * p<.05, **p<.01, ***p<.001

***2a. Study 2*** ***2b. Study 3*** ***2c. Study 4***

*n=124 n=122 n=129*

***Prosociality Prosociality Prosociality SDO***

*(Partial) (Partial) (Partial) (Partial)*

*CARING*  ***.346*** .252** .170*^p=.054^ -.431******

*.****281********.205**** ***.212* -.370******

*FAIRNESS* ***.263********.264***** *.105* ***-.512******

***.247********.229**** *.158* ***-.439******

*INGROUP .050 .009 .078* ***.262*****

*-.005 .046 .026 .144*

*AUTHORITY -.009 -.054 -.003* ***.379******

*-.064 -.018 -.072* ***.267*****

*PURITY .041 -.050 .097* ***.197****

*-.038 .002 .020 -.023*

**2a. Study 2**   **2b. Study 3 2c. Study 4**

n=112 (Exclusions) n=115 (Exclusions) n=117 (Exclusions)

CARING  **.202* .227* .188* -.415*****

.121 .164 **.214* -.346*****

FAIRNESS .137 **.241****  .095 **-.495*****

.109 **.210*** .136 **-.414*****

INGROUP .008 .035 .100 **.275****

-.021 .056 .053 .154

AUTHORITY -.067 -.040 -.006 **.416*****

-.093 -.028 -.081 **.279****

PURITY .013 -.043 .122 **.204***

-.038 -.023 .050 -.009

Notes. “Partial” refers to partial correlations with political orientation, religiosity, and gender controlled. Zero-order correlation coefficient is presented on top, partial correlation coefficient is underneath. SDO = Social Dominance Orientation. Boldface indicates significant correlations. * *p*<.05, ***p*<.01, ****p*<.001

**Study 4: n=129**

**Help Kin Not Help Kin Help Acquaint. Not Help Acquaint.**

(Partial) (Partial) (Partial) (Partial)

CARING .077 **-.274** .246** -.243****

.058 **-.276** .293*** -.270****

FAIRNESS .087 **-.283*** .208*** -.120

.070 **-.276** .264**** -.144

INGROUP -.052 -.032 **.199*** -.142

-.044 -.065 .167 -.133

AUTHORITY -.052 -.147 .068 -.086

-.044 **-.196*** .014 -.070

PURITY .044 -.098 **.240**** -.124

.085 **-.184*** **.243**** -.130

**Study 4: n=117 (12 Exclusions)**

CARING .079 **-.207* .256** -.195***

.066 **-.201* .318*** -.230***

FAIRNESS .135 **-.210* .184*** -.100

.133 **-.199* .254**** -.139

INGROUP -.011 -.030 **.232*** -.162

-.012 -.052 **.191*** -.131

AUTHORITY .030 -.120 .096 -.078

.034 -.162 .026 -.031

PURITY .101 -.106 **.270**** -.144

.131 -.167 **.268**** -.112

Notes. “Partial” refers to partial correlations with political orientation, religiosity, and gender controlled. Zero-order correlation coefficient is presented on top, partial correlation coefficient is underneath. Boldface indicates significant correlations. * *p*<.05, ** *p*<.01, *** *p*<.001

**References**

1. Caprara GV, Barbaranelli C, Pastorelli C, Cermak I, Rasza S (2001) Facing guilt: Role of negative affectivity, need for reparation, and fear of punishment in leading to prosocial behaviour and aggression. Eur J Pers 15: 219-237.

2. Cohen TR, Wolf ST, T. PA, Insko CA (2011) Introducing the GASP scale: A new measure of guilt and shame proneness. J Pers Soc Psychol 100: 947-966.

3. Silfver M, Helkama K, Lönnqvist J-E, Verkasalo M (2008) The relation between value priorities and proneness to guilt, shame, and empathy. Motiv Emot 32: 69-80.

4. McIlwain D (2011) Young Machiavellians and the traces of shame: Coping with vulnerability to a toxic affect. In: Barry C, Kerig P, Stellwagen K, Barry T, editors. Narcissism and Machievellianism in Youth: Implications for the Development of Adaptive and Maladaptive Behavior. Washington, DC: APA. pp. 213-231.

5. Graham J, Nosek BA, Haidt J, Iyer R, Koleva S, et al. (2011) Mapping the moral domain. J Pers Soc Psychol 101: 366-385.

6. Dahling JJ, Whitaker BG, Levy PE (2009) The development and validation of a new Machiavellianism scale. J Manage 35: 219-257.

7. Pratto F, Sidanius J, Stallworth LM, Malle BF (1994) Social dominance orientation: A personality variable predicting social and political attitudes. J Pers Soc Psychol 67: 741-763.
